# The use of mediation analysis to assess the effects of a behaviour change communication strategy on bed net ideation and household universal coverage in Tanzania

**DOI:** 10.1186/s12936-014-0531-0

**Published:** 2015-01-21

**Authors:** Emily E Ricotta, Marc Boulay, Robert Ainslie, Stella Babalola, Megan Fotheringham, Hannah Koenker, Matthew Lynch

**Affiliations:** Johns Hopkins Center for Communication Programs, Johns Hopkins Bloomberg School of Public Health, 111 Market Place Suite 310, Baltimore, MD 21202 USA; President’s Malaria Initiative, US Agency for International Development, Washington, DC USA

**Keywords:** Malaria, Mediation, Ideation, Social behaviour change communication, ITN

## Abstract

**Background:**

SBCC campaigns are designed to act on cognitive, social and emotional factors at the individual or community level. The combination of these factors, referred to as ‘ideation’, play a role in determining behaviour by reinforcing and confirming decisions about a particular health topic. This study introduces ideation theory and mediation analysis as a way to evaluate the impact of a malaria SBCC campaign in Tanzania, to determine whether exposure to a communication programme influenced universal coverage through mediating ideational variables.

**Methods:**

A household survey in three districts where community change agents (CCAs) were active was conducted to collect information on ITN use, number of ITNs in the household, and perceptions about ITN use and ownership. Variables relating to attitudes and beliefs were combined to make ‘net ideation’. Using an ideational framework, a mediation analysis was conducted to see the impact exposure to a CCA only, mass media and community (M & C) messaging only, or exposure to both, had on household universal coverage, through the mediating variable net ideation.

**Results:**

All three levels of exposure (CCA, M & C messaging, or exposure to both) were significantly associated with increased net ideation (CCA: 0.283, 95% CI: 0.136-0.429, p-value: <0.001; M & C: 0.128, 95% CI: 0.032-0.334, p-value: 0.018; both: 0.376, 95% CI: 0.170-0.580, p-value: <0.001). Net ideation also significantly increased the odds of having universal coverage (CCA_OR_: 1.265, 95% CI: 1.118-1.433, p-value: <0.001; M & C_OR_: 1.264, 95% CI: 1.117-1.432, p-value: <0.001, both_OR_: 1.260, 95% CI: 1.114-1.428, p-value: <0.001). There were no significant direct effects between any exposure and universal coverage when controlling for net ideation.

**Conclusions:**

The results of this study indicate that mediation analysis is an applicable new tool to assess SBCC campaigns. Ideation as a mediator of the effects of communication exposure on household universal coverage has implications for designing SBCC to support both mass and continuous distribution efforts, since both heavily rely on consumer participation to obtain and maintain ITNs. Such systems can be strengthened by SBCC programming, generating demand through improving social norms about net ownership and use, perceived benefits of nets, and other behavioural constructs.

## Background

Social and Behaviour Change Communication (SBCC) is used to strengthen health outcomes by disseminating targeted messages to communities that aim to improve health behaviours and reduce risk [1]. SBCC programmes are evidence-based and grounded in theory, and they promote behaviour change at multiple socio-ecological levels [2]. Dissemination can involve various channels, including radio, community events, and print media, and are therefore a good way to expose a large audience to health promotion messages [3]. In the malaria field, SBCC has been used successfully for promoting intermittent preventive treatment for malaria in pregnancy (IPTp), malaria treatment uptake, and insecticide-treated net (ITN) use [4-6].

SBCC programmes are particularly important for improving ITN ownership and use, because in addition to the problem of acquiring enough nets, decisions and actions at the individual or household level are necessary to maintain those nets. Once a net has been obtained, for example through a campaign, an antenatal care (ANC) visit or by purchasing a net in the market, there are a few decisions that must be made. These include the decision to keep, hang and use that net within the household rather than selling or giving the net away, ‘saving’ the net for later, or using it for other purposes. Behaviour around net care and repair also contributes to the number of usable nets in the household. While ITN distribution campaigns have proven to be an effective and cost-efficient means of increasing household net access and reducing malaria morbidity and mortality [7,8], lack of access to nets is still a significant challenge in achieving consistent net use [9,10]. To confuse the situation, ‘access’ can refer to very different concepts: either general, systems-level net supply in terms of households’ ability to obtain nets through distributions and private vendors, or as used in universal coverage indicators. As an indicator, ‘household access’ describes the proportion of *households* with enough nets (using the criterion one net for every two people) [11,12]. A separate indicator is ‘population access’ or ‘intra-household access’, the indicator that measures the proportion of *individuals* within the household that have access to a net, based on the number of nets the household owns and the number of household members, and assuming that one net protects two people [9,10]. Population access is demonstrated to have a close, linear relationship with net use, as having enough nets within a household is a primary driver of use by household members [13]. Given that household decisions are directly affecting the number of nets that households acquire and keep, using household universal coverage, defined as a household having one net for every two people [14], as an indicator may both capture the important aspect of intra-household access to a net, as well as the behaviours necessary to achieve and/or maintain enough nets in the household. Further, it is amenable to influence and change by SBCC campaigns, which are designed to act on cognitive, social and emotional factors at the individual or community level. The combination of these factors, which include beliefs and values, social norms, emotional responses, and social support and influence about a particular subject, is referred to as ‘ideation’ [15].

Ideation itself is defined as a new way of thinking which can be diffused through a population via social interaction [16], and while this paper demonstrates its use in malaria research for the first time, this concept is frequently used in family planning and HIV behavioural research to demonstrate the influence of these combined factors on uptake of contraception [17,18] and on safer sex behaviour [19,20]. The theoretical framework for the ideational model developed by Weber and Dumont in 1909 [17] and implemented by Kincaid [16,21] is that psychosocial variables play a role in determining intention and behaviour by reinforcing and confirming decisions [12,14]. This framework suggests that behaviour is due to an individual’s ideas or thoughts and attributes changes in behaviour to alterations in beliefs, typically via mass media exposure and social interaction [17]. Additionally, the model assumes that ideational variables can mediate the influence communication has on behaviour because the rate of diffusion of information is determined by the values and beliefs on a topic [16,17].

One way to evaluate the effect of ideational variables is through mediation analysis. Mediation analysis provides a way of understanding the mechanism relating an exposure variable to a specific outcome by identifying influential intermediate variables. By fitting a series of regression models to data containing a mediating variable, the effects of these intermediate variables can be computed [22,23].

This study introduces ideation theory and mediation analysis as a way to evaluate the impact of a malaria SBCC campaign in Tanzania. Malaria is a significant public health problem in Tanzania, with 93% of the population living at risk for disease and an estimated 70,000 deaths per year [24]. In recent years, Tanzania has made a considerable effort to promote use of ITNs through two mass distribution campaigns, which took place between 2009 and 2011 [25-27]. These campaigns increased the proportion of households that reported owning at least one ITN from 39% in 2007–2008 to over 90% in 2011 [13], and increased reported use by all *de facto* household members the night before the survey from 20% to almost 70% [13]. Additionally, Tanzania launched the Communication and Malaria Initiative in Tanzania (COMMIT) project in 2008, which used social mobilization and communication activities to reinforce messaging about malaria prevention and treatment [11].

The study had two objectives related to the use of mediation analysis as a viable method for evaluating SBCC programme impact for malaria. Working backwards to establish a plausible relationship, it first aimed to ascertain whether ‘household universal coverage’ was related to ideational variables surrounding net acquisition and use. The second objective was to determine the extent to which exposure to a communication programme influenced household universal coverage, mediated by the ideational variables mentioned above. These ideational variables surrounding universal coverage were measured through a household survey conducted in three regions in Tanzania where a multi-level SBCC campaign had been implemented.

## Methods

### Study context

The COMMIT project wasfunded by USAID under the President’s Malaria Initiative (PMI) and led by The Johns Hopkins Center for Communication Programs (CCP) in collaboration with Jhpiego, Population Services International (PSI), PMI and the Tanzania Ministry of Health and National Malaria Control Programme (NMCP) [11].

COMMIT was a multi-tiered SBCC project that aimed to foster positive social norms around malaria prevention and treatment and increase confidence among the population on the correct use of interventions such as ITNs, a concept known as self-efficacy. At the centre of the project was the Rural Communication Initiative that was implemented through more than 65 community-based organizations, and supervised 1,200 community change agents (CCAs) responsible for mobilizing communities around malaria prevention, treatment and control. Community-level activities were supported by reinforcement components, including mass media (via radio programming) and health provider strengthening to ensure individuals and families had the information, motivation and self-efficacy to put into practice appropriate malaria prevention and treatment behaviour.

The CCAs began mobilizing communities in late 2008 around malaria prevention, treatment and control. Each CCA was responsible for a ward (the lowest administrative unit), which was comprised of between four and six villages, and ranged in size from 10–20 km. CCAs were selected from one of the villages by community leaders and knew the communities well. CCA activities and messaging focused on promoting malaria prevention and treatment-seeking behaviours, including the importance of using ITNs, prevention of malaria during pregnancy, and preparing communities to accept indoor residual spraying (IRS) for vector control in IRS-targeted areas. Through village talks, school campaigns and health clinic discussions, the CCA engaged the community in discussions around malaria prevention and treatment. The model used was the Communication for Social Change model that leads communities from collective dialogue to collective action. The expected result was that communities develop action plans to ensure that all households have sufficient access to ITNs, and that all pregnant women attend ANC and receive sulphadoxine-pyrimethamine (SP) for IPTp. Through their mobilization activities, CCAs reinforced the mass media messages on malaria prevention or treatment. All of these activities were designed to contribute to families and communities adopting new social norms, such as consistent net use by all members of the population or malaria diagnostic testing for fevers.

From 2009 and 2011, Tanzania also implemented two mass long-lasting insecticidal bed net (LLIN) distribution campaigns; the first targeted children under five year old [26], while the second, a universal coverage campaign (UCC), aimed to deliver one net for every two people in a household [27]. Concurrently, the Tanzania National Voucher Scheme (TNVS) was operational and delivered ITNs to pregnant women and infants via a voucher system.

### Data collection

**Figure 1:**
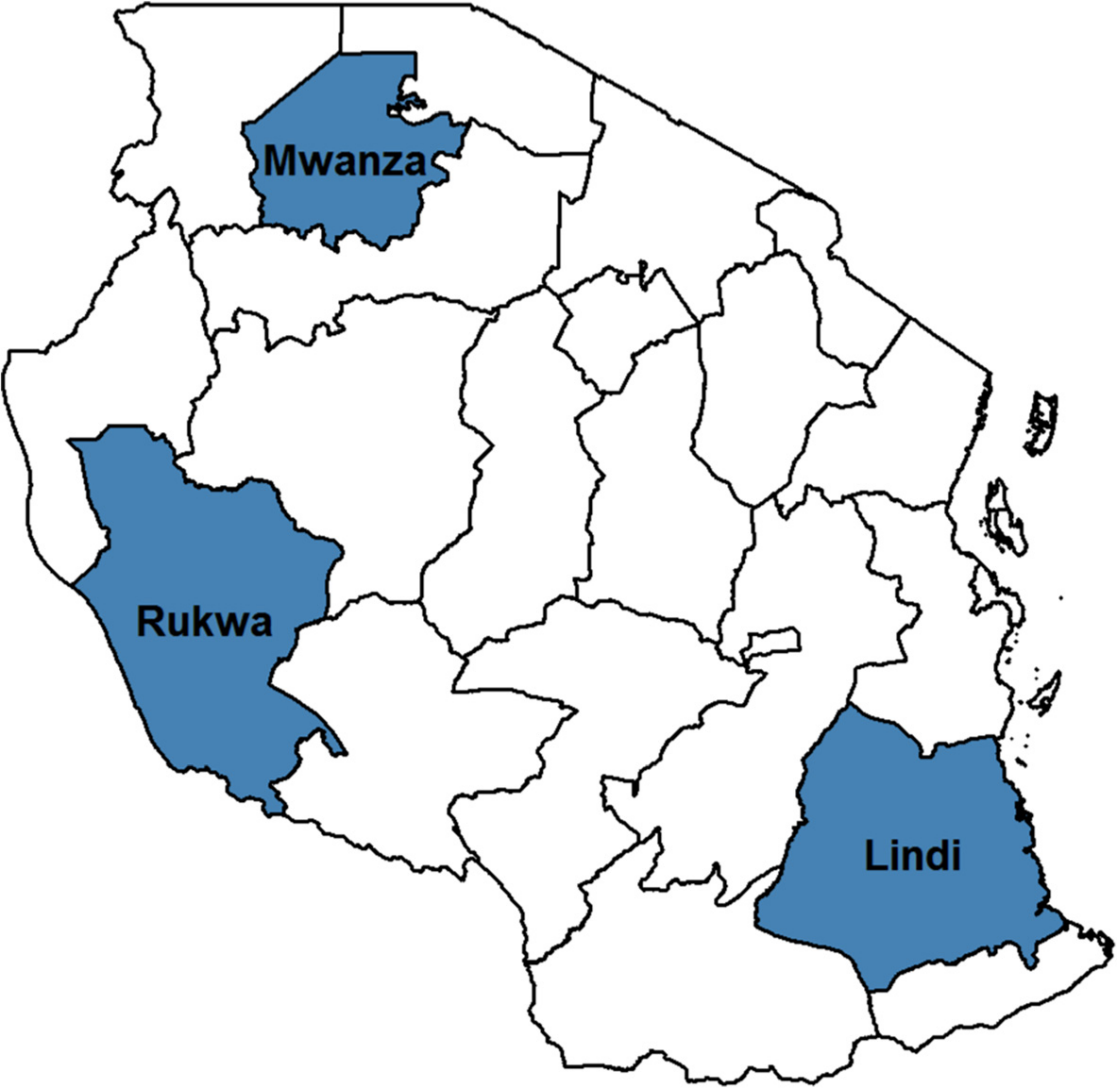
**Map of study sites for the community change agent assessment.**

A household survey was conducted in October and November 2011 to collect data in three regions of Tanzania with differing levels of reported CCA activity. Monitoring reports from CCAs over a three-month period indicated that CCA activities were high in Mwanza (greater than eight activities per agent), moderate in Rukwa (four to eight activities per agent), and low in Lindi (less than four activities per agent) (Figure 1).

In each region three to five wards were selected using simple random sampling, with 60 community clusters (villages) enumerated and then made eligible for selection from each. A household was considered eligible and one adult household member was selected for the survey if they met the following criteria: 1) had slept in the household for at least five nights in the previous week; 2) had a child under five years of age living in the household; 3) agreed to a follow-up visit by study staff; and, 4) could inform on their level of interaction with CCAs in their communities. A total of 1,142 household members were interviewed. A structured questionnaire was used to collect data on basic demographics, specific household interactions with the CCAs, as well as beliefs about malaria and ITN use. Questions were also asked about perceived community attitudes toward ITNs and their perception of CCA involvement in the general area. Indices were constructed from four to eight Likert-scaled questions each in the questionnaire. Likert scales were used to assess whether respondents ‘strongly agreed’, ‘agreed’, ‘disagreed’, or ‘strongly disagreed’, and these values were then recoded after data entry and cleaning into a binomial variable of agree or disagree, with no differences between strongly agreed and agreed.

### Variables

The main outcome was binary household universal coverage. If households had at least 1 ITN per 2 people, they were categorized as having universal coverage, while any household below this threshold was considered to not have universal coverage.

A composite variable called ‘net ideation’ was created by adding together respondent scores on four sub-indices for key malaria ideational constructs: perceived social norms surrounding net ownership and use, belief in their own ability to use nets properly (self-efficacy), belief that nets are an effective means of preventing malaria (sometimes termed ‘response efficacy’), and perceived threat of malaria. The values for this additive index ranged from 0–4; the higher the net ideation value, the more positive the individual felt about nets both personally and within the community. There were three exposure levels of interest in this study: exposure to media and community (M & C) messaging, exposure to a CCA, or exposure to both (CCA and M & C messaging). Exposure to a CCA included any reported contact with a CCA, ranging from receiving home visits by a CCA to attending group talks, public meetings, cultural shows, or school events. Exposure to M & C messaging included any reported exposure to messages through both mass media (radio spots and discussion programmes, TV spots, printed materials) and community channels (‘mid media’, including mobile video units and road shows). Mass media and community messaging were combined into one exposure (M & C messaging) because more of an effect was seen when exposed to both than either communication channel on its own.

### Data analysis

Data were analysed using R: A Language and Environment for Statistical Computing (R Foundation for Statistical Computing v3.0.1 – Good Sport, Spring 2013, Vienna, Austria), and Stata/SE 13.1 for Windows (StataCorp. 2013. Stata Statistical Software: Release 13. College Station, TX, USA: StataCorp LP). Mediation analysis was conducted as described by Baron and Kenny [28] and MacKinnon [23,29]. Typical mediation is done in three steps: 1) testing the direct relationship between the exposure (CCA, M & C messaging, or exposure to both) on the outcome (household universal coverage); 2) testing the effect of the exposure on the mediating variable (net ideation), again controlling for other variables; and, 3) relating the mediating variable to the outcome after controlling for the exposure and other variables. Step 1 provides the *direct effect* of the exposure on the outcome, after controlling for the mediating variable. In mediation analysis with continuous predictors, the product of the estimates from steps 2 and 3 provide the *indirect effect* of exposure on the outcome, also known as the amount of mediation provided by the mediator (net ideation) in the relationship between the exposure and the outcome. The direct effect plus the indirect effect produce the total effect. The proportion mediated can be obtained by dividing the indirect effect by the total effect. However, due to the fact that the exposure and outcome are both dichotomous in this analysis, resulting in two different estimation scales, this decomposition of effects does not accurately estimate the effect sizes [30]. Instead, the estimated coefficients must be scaled to make them comparable across equations [30], which the ‘mediation’ package in the R statistical software does automatically, resulting in probability estimates [22].

This study assessed the effect that varying levels of exposure to a behaviour-change programme (CCA, M & C messaging, or exposure to both) had on whether a household had universal coverage, through changes to net ideation. Multilevel regressions were run with a random intercept for ward (to control for inter-ward variation in CCA activity and diffusion of M & C messaging within a community). Additional covariates were selected using backward stepwise elimination by Akaike Information Criterion. Variance was estimated using a quasi-Bayesian Monte Carlo approach with 1000 simulations. The selected variables were level of education (none/incomplete, primary, higher), region of residence, and SES quintile (a composite variable consisting of multiple standard economic questions). Only single-mediator analysis was conducted in this study.

### Ethical approval

This study was approved by the Johns Hopkins University Institutional Review Board and the National Institute of Medical Research in Dar es Salaam.

## Results

A total of 260 males and 882 females were interviewed, with ages ranging from 18 to 96 years old and a median age of 30 years. In total, 313 respondents (27%) reported exposure to a CCA, whether through household visits or community events held by a CCA. A total of 282 respondents (25%) reported exposure to M & C messaging, and 124 (12%) reported exposure to both channels. Only 117 (10%) were unexposed.

### ITN ownership

**Figure 2:**
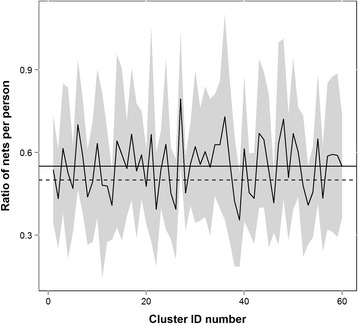
**Number of ITNs per person by cluster (net ratio).** Black line is the mean number of ITNs per person in a cluster, grey is +/− 1 SD from the mean. The solid horizontal line is the overall mean (0.55 ITNs/person) and the dashed horizontal line is the WHO goal for universal coverage (0.50 ITNs/person).

The average number of ITNs per person in a household all 60 clusters was 0.55 ITN per person, and this ranged from 0.09 to 2.00 ITNs per person per cluster. This average compares favourably to the WHO definition of universal coverage [14]. Figure 2 shows the variability in the number of ITNs per person (net ratio) by village (ICC: 0.106), and demonstrates that within village variance is large in most cases (black line indicates village mean, grey shading +/− 1 SD from the mean). Net ratio also differed by region (ICC: 0.062), with Lindi having an average of 0.62 nets per person, Mwanza having 0.55 nets per person, and Rukwa having 0.49 nets per person (p-value: <0.001). In Lindi, 76% of households had universal coverage, while in Mwanze it was 62% and in Rukwa, 50%.

### Mediation analysis

**Figure 3:**
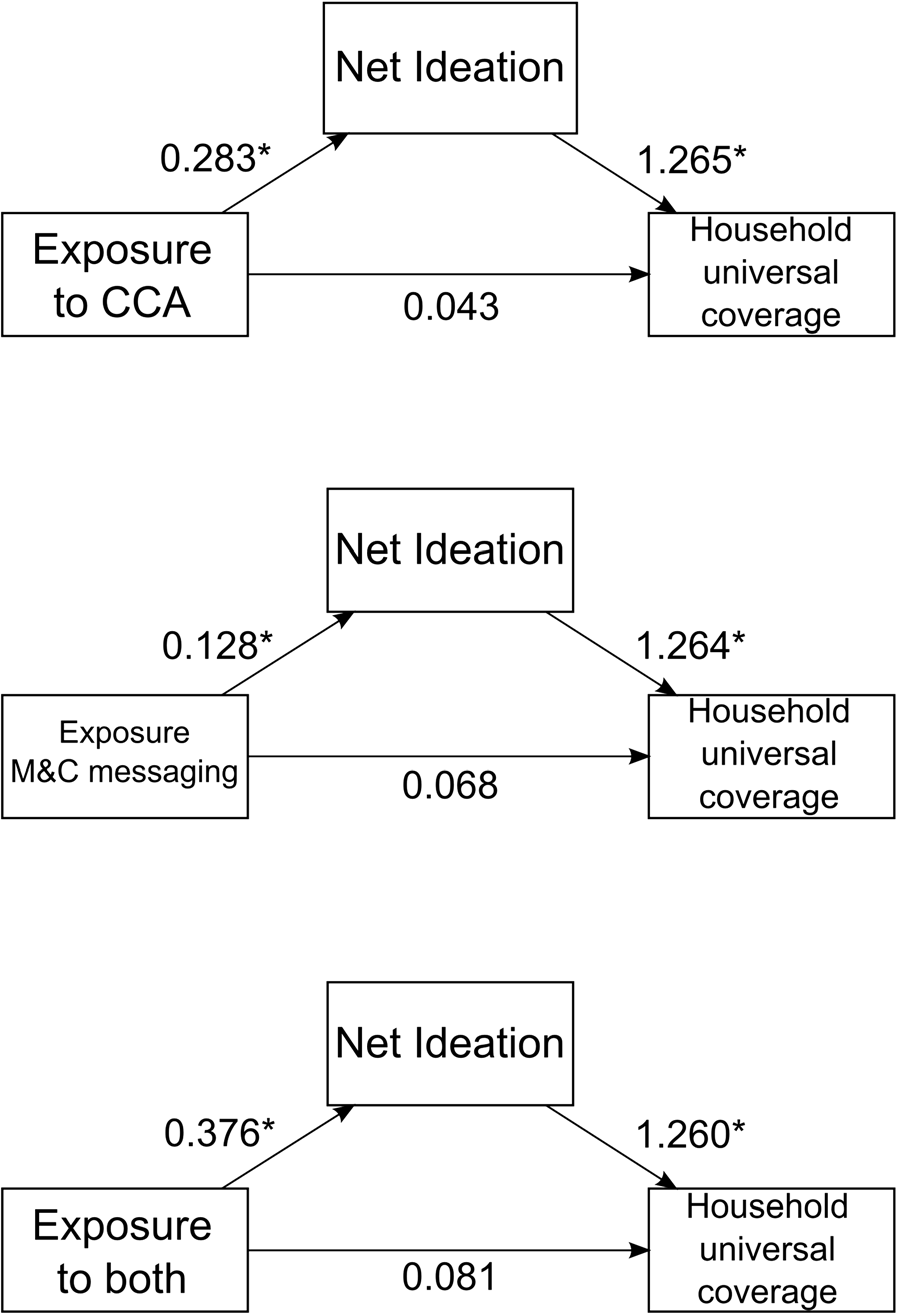
**Mediation pathways for each category of exposure**. Estimate for each section of the pathway is indicated. Linear regressions coefficients presented for the relationship between exposure and mediator, and odds ratios are presented for the relationship between the mediator and the outcome. *p ≤ 0.05.

To assess the relationship between ideational variables related to household universal coverage, the variable net ideation was created. When controlling for BCC exposure, net ideation significantly increased the odds of a household having universal coverage by roughly 25% (CCA_OR_: 1.265, 95% CI: 1.118-1.433, p-value: <0.001; M & C_OR_: 1.264, 95% CI: 1.117-1.432, p-value: <0.001, both_OR_: 1.260, 95% CI: 1.114-1.428, p-value: <0.001) (Figure 3). Next, the association between the exposure variables and net ideation was assessed (Figure 3). Any exposure, either to a CCA, M & C messaging or both, significantly increased net ideation (CCA: 0.283, 95% CI: 0.136-0.429, p-value: <0.001; M & C: 0.128, 95% CI: 0.032-0.334, p-value: 0.018; both: 0.376, 95% CI: 0.170-0.580, p-value: <0.001). While small, all exposure categories had a significant indirect effect on household universal coverage, through net ideation, which accounted for 11 to 23% of the effect of these exposures on household universal coverage (CCA: 0.014, 95% CI: 0.005-0.024, p-value: <0.001; M & C: 0.009, 95% CI: 0.001-0.018, p-value: 0.02; both: 0.017, 95% CI: 0.006-0.032, p-value: <0.001) ITN access (not shown in figure). There were no significant direct effects between outcome and household universal coverage when controlling for net ideation (Figure 3). The total effects for exposure to M & C messaging or both were significant (M & C: 0.075, 95% CI: 0.011-0.138, p-value: 0.03; both: 0.100, 95% CI: 0.006-0.180, p-value: 0.03).

## Discussion

While other studies have shown effects of BCC on specific behaviour [3,5,31], this study is the first to make the link between exposure to BCC messaging and increased ownership of ITNs within households through the mediating effect of ideation. Ensuring household universal coverage and increasing use is imperative for effective control and prevention of malaria, and while making ITNs available via ANC, expanded programme on immunization (EPI), mass campaigns, and other channels such as schools, community distributors and retail sector is critical, programme planners must also consider the decisions and behaviour leading to net acquisition, retention, maintenance, and use within a household.

Most often, nets are distributed to households via mass campaigns or at ANC/EPI visits, aimed at providing equitable access to all households. For example, the under-five catch-up campaign and the universal coverage campaign in Tanzania were highly equitable in their net distributions [26,27], however, given the variation seen in the number of ITNs per person per household within villages in this study (Figure 2), the campaigns and voucher system did not guarantee that households had enough nets. As distance to a health facility or exposure to a distribution campaign is similar across households, it is reasonable to assume comparable net ratios among homes in the same cluster if geographic access to an ITN was the main driver of universal coverage. As this is not the case, it invites discussion about other factors influencing whether a household has universal coverage, including household size or the implementation of the campaign, but also behavioural and decision-making variables both within a household and in a cluster, and whether these pose a significant barrier to acquiring and keeping ITNs.

In Tanzania, ANC and EPI distributions have a further level of decision-making required, as nets are not distributed directly to recipients at the health clinic. Instead, under the TNVS, health care providers issue pregnant women with vouchers (at ANC visits) and infant vouchers (at EPI visits), which the women or caretakers can then redeem at a nearby retail point for nets at a significantly reduced price, a process which is described in detail elsewhere [32-36]. At the time of the survey, the top-up amount for TNVS was 500 TZS [25]. For mass campaigns, household decision-making can affect participation in the registration activities, where campaign workers visit households, household members are counted, and the number of nets the household needs is recorded. Households can choose whether or not to allow the registration teams into their home, or to ensure that someone is home to meet them when the family may be otherwise working or away from home. However, because over 90% of households were registered during the Tanzania UCC either by being present at home, by visiting a registration centre, or by visiting an executive office if no one was present, the proportion missing registration was limited [27]. Once registered, the household could then decide to participate or not in the issuing of the ITNs at the nearest distribution point. Once the net arrives at home, household decision-making around net care will influence the maintenance and use of nets in useable condition.

This study aimed to use mediation analysis and ideational theory to assess the relationship between variables relating to the acquisition and ownership of ITNs, and having sufficient ITNs per person in a household. This framework has been applied commonly in reproductive health and family planning to evaluate the efficacy of communication campaigns [16-18], and is just as applicable to malaria SBCC. The results show a significant relationship between net ideation and household universal coverage, with an increase in the likelihood of a household having universal coverage associated with higher levels of net ideation. This suggests that an individual’s beliefs influence the number of ITNs per person in the household, and that people with a more positive net ideation are more likely to have achieved universal coverage within their household.

Additionally, this study evaluated the relationship between exposure to the SBCC campaign (through CCA activities, M & C messaging, or exposure to both), via net ideation, to household universal coverage. In this setting, all exposures were significantly related to an increase in the likelihood of achieving universal coverage, and this relationship was mediated through net ideation. No direct effect of any exposure on universal coverage was observed when controlling for net ideation. The proportion of the relationship between the exposure and household universal coverage was only mediated by net ideation 11 to 23%, indicating that there are additional unmeasured pathways or ideational variables that play a role in the number of ITNs per person in a household. These could include decisions to obtain a net, maintaining nets in usable condition, as well as decisions on discarding or when to stop using a net. More research is needed to determine how different ideational variables contribute to the likelihood of a household achieving universal coverage, and indeed, new ways of measuring these and other socio-behavioural variables are required to get an accurate picture of their influence in net acquisition and use.

One limitation of this study was that CCAs were required to self-report their activities at a centralized location and some were unable to travel there as frequently as others. This could have led to inconsistencies in the reported data, resulting in less accurate distinctions between high, moderate and low activity regions. Additionally, the CCA programme was not rolled out in all regions at the same time, resulting in variable levels of reported activity throughout the study period. Due to the high level of exposure in the study area, the control groups for each exposure category were not exclusively households unexposed to all CCA activity or messaging. For example, individuals who were unexposed to a CCA might still have reported exposure to M & C messaging. While this limits the ability to attribute the increase seen in net ideation and universal coverage to any particular channel, it does provide a good starting point for using this methodological approach to evaluate the impact of SBCC campaigns for malaria. Finally, the effect sizes seen in this study, particularly that of the indirect effect of exposure on universal coverage, were small. However, it is important to remember that when multiplied over a large population, these effects can still contribute significantly to an increase in positive attitudes, beliefs, and practices surrounding malaria prevention.

## Conclusion

The results of this study indicate that a malaria SBCC campaign, relying on a combination of media and community messaging and CCAs who act as drivers of information within the community, can be effective at influencing individual perceptions and beliefs about ITNs, in turn increasing the likelihood of a household achieving universal coverage. It also demonstrates the usefulness of evaluating such campaigns by assessing ideational variables and how these mediate the relationship between campaign exposure and a behavioural outcome. Ideation as a mediator of the effects of communication exposure on universal coverage has implications for designing SBCC to support both mass and continuous distribution efforts, since both heavily rely on consumer participation to obtain and maintain ITNs. Such systems can be strengthened by SBCC programming, generating demand through improving social norms about net ownership and use, perceived benefits of nets, and other behavioural constructs.

## References

[1] Defining Social and Behavior Change Communication (SBCC) and Other Essential Health Communication Terms. Washington, DC: The Manoff Group; 2012:1–4.

[2] Vaughan PW, Rogers EM (2000). A staged model of communication effects: evidence from an entertainment-education radio soap opera in Tanzania. J Health Commun.

[3] Koenker H, Keating J, Alilio M, Acosta A, Lynch M, Nafo-Traore F (2014). Strategic roles for behaviour change communication in a changing malaria landscape. Malar J.

[4] Desrochers RE, Siekmans K, Berti PR, Bramhill K, Buchan SAW, Battah GK, Gbetoglo D, Vignikin K, Sabino A (2014). Effectiveness of post-campaign, door-to-door, hang-up, and communication interventions to increase long-lasting, insecticidal bed net utilization in Togo (2011–2012): a cluster randomized, control trial. Malar J.

[5] Gies S, Coulibaly SO, Ky C, Ouattara FT, Brabin BJ, D’Alessandro U (2009). Community-based promotional campaign to improve uptake of intermittent preventive antimalarial treatment in pregnancy in Burkina Faso. Am J Trop Med Hyg.

[6] Boulay M, Lynch M, Koenker H (2014). Comparing two approaches for estimating the causal effect of behaviour-change communication messages promoting insecticide-treated bed nets: an analysis of the 2010 Zambia malaria indicator survey. Malar J.

[7] Alba S, Nathan R, Schulze A, Mshinda H, Lengeler C (2014). Child mortality patterns in rural Tanzania: an observational study on the impact of malaria control interventions. Int J Epidemiol.

[8] Yukich JO, Lengeler C, Tediosi F, Brown N, Mulligan J-A, Chavasse D, Stevens W, Justino J, Conteh L, Maharaj R, Erskine M, Mueller DH, Wiseman V, Ghebremeskel T, Zerom M, Goodman C, McGuire D, Urrutia JM, Sakho F, Hanson K, Sharp B (2008). Costs and consequences of large-scale vector control for malaria. Malar J.

[9] Hetzel MW, Gideon G, Lote N, Makita L, Siba PM, Mueller I (2012). Ownership and usage of mosquito nets after four years of large-scale free distribution in Papua New Guinea. Malar J.

[10] Eisele TP, Keating J, Littrell M, Larsen D, Macintyre K (2009). Assessment of insecticide-treated bednet use among children and pregnant women across 15 countries using standardized national surveys. Am J Trop Med Hyg.

[11] Communication Impact: Rural Communication Activities Increase Net Use in Tanzania. Baltimore, MD: Johns Hopkins Center for Communication Programs; 2010.

[12] MEASURE Evaluation, MEASURE DHS, President’s Malaria Initiative, Roll Back Malaria Partnership, UNICEF, World Health Organization: Household Survey Indicators for Malaria Control. 2013

[13] Koenker H, Kilian A (2014). Recalculating the net use gap: a multi-country comparison of ITN use versus ITN access. PLoS One.

[14] Global Malaria Programme: WHO Recommendations for Achieving Universal Coverage with Long-Lasting Insecticidal Nets in Malaria Control. Volume 2013. Geneva; 2014. (September 2013).

[15] Health Communication Partnership: Research 101 - A Primer for Health Communication Professionals: Ideation. Baltimore, MD: Johns Hopkins Center for Communication Programs; 2004.

[16] Kincaid DL (2000). Mass media, ideation, and behavior: a longitudinal analysis of contraceptive change in the Philippines. Commun Res.

[17] Babalola S, Vondrasek C (2005). Communication, ideation and contraceptive use in Burkina Faso: an application of the propensity score matching method. J Fam Plann Reprod Health Care.

[18] Babalola S, Vondrasek C, Brown J, Traore R, Babalola BS (2001). The impact of a Regional Family Planning Service Promotion in Sub-Saharan Initiative Africa: Evidence from Cameroon. Int Fam Plan Perspect.

[19] Nguyen HV, Le GM, Nguyen SM, Tran MN, Ha NM (2012). The effect of participatory community communication on HIV preventive behaviors among ethnic minority youth in central Vietnam. BMC Public Health.

[20] Babalola S, Awasum D, Quenum-renaud B (2002). The correlates of safe sex practices among Rwandan youth: a positive deviance approach. Afr J AIDS Res.

[21] Kincaid DL (2000). Social networks, ideation, and contraceptive behavior in Bangladesh: a longitudinal analysis. Soc Sci Med.

[22] Imai K, Keele L, Tingley D (2010). A general approach to causal mediation analysis. Psychol Methods.

[23] Mackinnon DP (2008). Introduction to Statistical Mediation Analysis.

[24] Gosoniu L, Msengwa A, Lengeler C, Vounatsou P (2012). Spatially explicit burden estimates of malaria in Tanzania: bayesian geostatistical modeling of the malaria indicator survey data. PLoS One.

[25] Koenker HM, Yukich JO, Mkindi A, Mandike R, Brown N, Kilian A, Lengeler C (2013). Analysing and recommending options for maintaining universal coverage with long-lasting insecticidal nets: the case of Tanzania in 2011. Malar J.

[26] Bonner K, Mwita A, McElroy PD, Omari S, Mzava A, Lengeler C, Kaspar N, Nathan R, Ngegba J, Mtung’e R, Brown N (2011). Design, implementation and evaluation of a national campaign to distribute nine million free LLINs to children under five years of age in Tanzania. Malar J.

[27] Renggli S, Mandike R, Kramer K, Patrick F, Brown NJ, McElroy PD, Rimisho W, Msengwa A, Mnzava A, Nathan R, Mtung’e R, Mgullo R, Lweikiza J, Lengeler C (2013). Design, implementation and evaluation of a national campaign to deliver 18 million free long-lasting insecticidal nets to uncovered sleeping spaces in Tanzania. Malar J.

[28] Baron R, Kenny D (1986). The moderator-mediator variable distinction in social psychological research: conceptual, strategic, and statistical considerations. J Pers Soc Psychol.

[29] Mackinnon DP, Fairchild AJ, Fritz MS (2010). Mediation analysis. Annu Rev Psychol.

[30] Mackinnon DP, Dwyer JH (1993). Estimating mediated effects in prevention studies. Eval Rev.

[31] Conteh L, Stevens W, Wiseman V (2007). The role of communication between clients and health care providers: implications for adherence to malaria treatment in rural Gambia. Trop Med Int Health.

[32] Mulligan J-A, Yukich J, Hanson K (2008). Costs and effects of the Tanzanian national voucher scheme for insecticide-treated nets. Malar J.

[33] De Savigny D, Webster J, Agyepong IA, Mwita A, Bart-Plange C, Baffoe-Wilmot A, Koenker H, Kramer K, Brown N, Lengeler C (2012). Introducing vouchers for malaria prevention in Ghana and Tanzania: context and adoption of innovation in health systems. Health Policy Plan.

[34] Gingrich CD, Hanson KG, Marchant TJ, Mulligan J-A, Mponda H (2011). Household demand for insecticide-treated bednets in Tanzania and policy options for increasing uptake. Health Policy Plan.

[35] Hanson K, Nathan R, Marchant T, Mponda H, Jones C, Bruce J, Stephen G, Mulligan J, Mshinda H, Schellenberg JA (2008). Vouchers for scaling up insecticide-treated nets in Tanzania: methods for monitoring and evaluation of a national health system intervention. BMC Public Health.

[36] Mushi a K (2003). Targeted subsidy for malaria control with treated nets using a discount voucher system in Tanzania. Health Policy Plan.

